# SOX4对非小细胞肺癌细胞A549的顺铂耐药作用的影响

**DOI:** 10.3779/j.issn.1009-3419.2017.05.12

**Published:** 2017-05-20

**Authors:** 维 李, 旭 刘, 国倩 张, 琳琳 张

**Affiliations:** 1 300193 天津，天津中医药大学第一附属医院检验科 Department of Clinical Laboratory, the First Teaching Hospital of Tianjin University of Traditional Chinese Medicine, Tianjin 300193, China; 2 300052 天津，天津医科大学总医院肿瘤科 Department of Oncology, Tianjin Medical University General Hospital, Tianjin 300052, China

**Keywords:** 肺肿瘤, A549, SOX4, 耐药性, Wnt信号通路, Lung neoplasms, A549, SOX4, Drug resistance, Wnt pathway

## Abstract

**背景与目的:**

肺癌是最严重的恶性肿瘤之一，其中非小细胞肺癌发病率在肺癌中居首位。部分患者对顺铂耐受是导致化疗失败的主要原因。SOX4是转录调节因子，在多种肿瘤的发生发展过程中发挥着重要的作用，通过调控β-catenin的表达对Wnt信号通路进行调控。本研究旨在探讨SOX4对非小细胞肺癌细胞A549的顺铂耐药作用的影响。

**方法:**

体外诱导法建立顺铂耐药的肺癌细胞株A549/DDP，CCK8法检测A549及A549/DDP细胞对顺铂的耐药性。绘制A549与A549/DDP细胞生长曲线。Western blot检测耐药细胞株中SOX4的蛋白表达水平。CCK8法检测敲减SOX4后耐药细胞株A549/DDP对顺铂耐药性。实时定量PCR和免疫印迹法检测敲减SOX4后A549/DPP细胞中β-catenin和Survivin蛋白的表达。

**结果:**

成功构建非小细胞肺癌顺铂耐药细胞株A549/DDP，其耐药性是亲本细胞的13.7倍，差异有统计学意义（*P* < 0.001）。生长曲线显示耐药细胞株与亲本细胞增殖速度没有统计学差异（*P* < 0.05）；与A549细胞相比，耐药细胞A549/DDP细胞中SOX4的表达显著增高（*P* < 0.001）。敲减SOX4后，A549/DDP细胞的耐药性显著降低；敲减SOX4后，A549/DDP细胞中β-catenin和Survivin的mRNA和蛋白表达水平显著降低。

**结论:**

SOX4的表达水平影响非小细胞肺癌细胞A549对顺铂的耐药性。

## Abstract (en)

**Background and objective:**

Lung cancer is one of the most serious disease and the incidence of nonsmall cell lung cancer (NSCLC) is the highest in lung cancer. The main reason for the failure of chemotherapy is the tolerance to cisplatin. Transcriptional regulator SOX4 plays an important role in the occurrence and development of many tumors, and regulates Wnt signaling pathway by regulating the expression of β-catenin. We aimed to investigate the role of SOX4 on cisplatin-resistance in NSCLC cell A549 cell.

**Methods:**

The cisplatin-resistance lung cancer cell line A549/DDP was constructed by induction method *in vitro*, and cisplatin-resistance detected by CCK8 assay. Growth curves of A549 and A549/DDP was calculated. The expression level of SOX4 in A549 and A549/DDP cells were detected by Western blot. A549/DDP were knockdown of SOX4 by siRNA transfection, and the cisplatin-resistance of detected by CCK-8 assay, the expression level of β-catenin and Survivin were detected by real-time PCR and Western blot.

**Results:**

The cisplatin-resistance cell line A549/DDP was constructed successfully, and its cisplatin-resistance is 13.7 times higher than in A549. There was no significance difference between A549 and A549/DDP in cell proliferation. The expression level of SOX4 is higher in A549/DDP than in A549. The cisplatin-resistance significantly decreased in A549/DDP cells after knockdown of SOX4 by siRNA transfection. The expression level of β-catenin and Survivin significantly decreased in A549/DDP cells after knockdown of SOX4.

**Conclusion:**

SOX4 can strengthen cisplatin-resistance of non-small cell lung cancer cell A549.

肺癌是近年来全球范围内发病率和死亡率最高的恶性肿瘤之一，其发病率和死亡率近年来也明显上升^[1, 2]^。肺癌中约85%为非小细胞肺癌（non-small cell lung cancer, NSCLC）^[3]^，其发病率在肺癌中居首位^[4]^，患者5年内生存率不足15%^[5]^。以顺铂（DDP）为主的联合化疗是目前治疗晚期NSCLC的标准方案，但目前部分患者对顺铂耐受是导致化疗失败的主要原因^[6]^。因此，对顺铂耐药的分子机制的研究对NSCLC的防治和提高疗效具有深远意义。SOX4是转录调控分子家族SOX重要成员，研究表明，SOX4在多种肿瘤的发生发展过程中发挥着重要的作用, 机制之一可能是其通过调控β-catenin的表达对Wnt信号通路进行调控^[7]^，而β-catenin在NSCLC细胞对顺铂的耐受中起着重要作用^[8]^。因此，本研究旨在研究SOX4对小细胞肺癌细胞A549的顺铂耐药作用及其分子机制。

## 1 材料及方法

### 1.1 材料

1640培养基、小牛血清购于美国HyClone公司。顺铂（DDP）为齐鲁制药厂生产。SOX4的siRNA购自QIAGEN公司。SOX4、β-catenin和Survivin抗体购自Abcam公司。免疫印迹化学发光系统购自Syngene公司。MTT购自Sigma公司。Trizol、SYBR和Lipo2000购自美国Invitrogen公司。RevertAid First Strand cDNA Synthesis Kit购自加拿大Fermentas公司。肺癌细胞株A549由本实验室保存。

### 1.2 方法

#### 1.2.1 细胞培养

肺癌细胞A549培养于含10%FBS的1640培养基中，培养液含青霉素/链霉素100 U/mL，将细胞置于37 ℃、5%CO_2_培养箱中培养。

#### 1.2.2 体外诱导法建立顺铂耐药的肺癌细胞株A549/DDP

对数生长期A549细胞在含有0.1 μM顺铂的1640培养基培养4周后，将细胞消化传代用正常培养基培养，待细胞贴壁后，将顺铂浓度提高至0.2 μM，培养4周；再依次将药物浓度提高到0.4 μM、0.6 μM、0.8 μM、1 μM……2 μM培养。在2 μM浓度下维持培养，初步得到A549/DDP细胞。

#### 1.2.3 A549细胞与耐药A549/DDP细胞细胞生长曲线测定

取对数生长期的A549细胞和耐药A549/DDP细胞，以5×10^3^个/孔接种于24孔板中，100 μL/孔，37 ℃、5%CO_2_培养，分别于24 h、48 h、72 h、96 h和120 h胰酶消化，对细胞进行计数，每组3个复孔，每孔计数3次，取平均值绘制细胞生长曲线。

#### 1.2.4 CCK8检测A549及A549/DDP细胞对顺铂耐药性

取对数生长期的A549细胞和耐药A549/DDP细胞，以0.3×10^5^个/mL接种于96孔板中，100 μL/孔，37 ℃、5%CO_2_培养。20 h后，加入梯度浓度的顺铂处理，每组设置3个复孔，培养48 h后，将旧培养基吸去，置换含10%CCK-8的新鲜培养基，37 ℃、5%CO_2_继续培养，3 h后，测450 nm吸光度值，计算半抑制浓度（half maximal inhibitory concentration, IC_50_）。

#### 1.2.5 A549/DDP细胞SOX4 siRNA转染

取对数生长期的A549/DDP细胞，以1×10^6^个/mL接种于六孔板中，1 mL/孔，37 ℃、5%CO_2_培养。24 h后用Lipo2000将SOX4的两条siRNA以及siRNA con转染A549/DDP细胞中，转染48 h后检测A549/DDP细胞对顺铂半数抑制浓度IC_50_，方法同1.2.4。

#### 1.2.6 实时荧光定量PCR检测mRNA表达水平

用Trizol法提取非小细胞肺癌细胞A549/DDP的总RNA，利用RevertAid First逆转录试剂盒得到cDNA，以cDNA为模板，分别以SOX4、β-catenin及Survivin实时定量PCR引物进行RT-PCR反应。SOX4引物：上游引物：5’-GGCCTGTTTCGCTGTCGGGTC-3’；下游引物：5’-GCCTGCATGCAACAGACTGGCA-3’；β-catenin引物：上游引物：5’-TGGTGCCCAGGGAGAACCC-3’；下游引物；5’-GTCAGCACCAGGGTGGTGGCA-3’；Survivin引物：上游引物：5’-TGGCCCAGTGTTTCTTCTGCTTCA-3’；下游引物：5’-AAAGGAAAGCGCAACCGGACGAAT-3’；内参GAPDH引物：上游引物：5’-GAAGGTGAAGGTCGGAGTC-3’；下游引物：5’-GAAGATGGTGATGGGATTTC-3’。实验重复3次。

#### 1.2.7 Western blot检测蛋白表达

水平收集细胞，用细胞裂解液RIPA裂解提取总蛋白。SDS-PAGE凝胶电泳分离，恒流300 mA转移至PVDF印迹膜。5%脱脂牛奶封闭1 h后，加入一抗，4 ℃孵育过夜。次日PBST洗膜，二抗室温孵育1 h。用化学发光法显色，凝胶成像系统采集成像。

### 1.3 统计学分析

应用SPSS 17.0软件进行统计学处理，结果用Mean±SD描述，组间比较应用*t*检验，以*P* < 0.05为有统计学差异。

## 2 结果

### 2.1 A549及A549/DDP细胞对顺铂耐药性

本研究检测了A549/DDP细胞及其亲本细胞A549对顺铂的半数抑制浓度IC_50_，结果如图 1所示，A549/DDP细胞对顺铂耐药性显著增强，A549/DDP和A549细胞的IC50分别为（27.87±0.92）μM和（2.04±0.37）μM，两者具有显著差异（*P* < 0.001）。耐药细胞株对顺铂耐药性增加了13.7倍。

**1:**
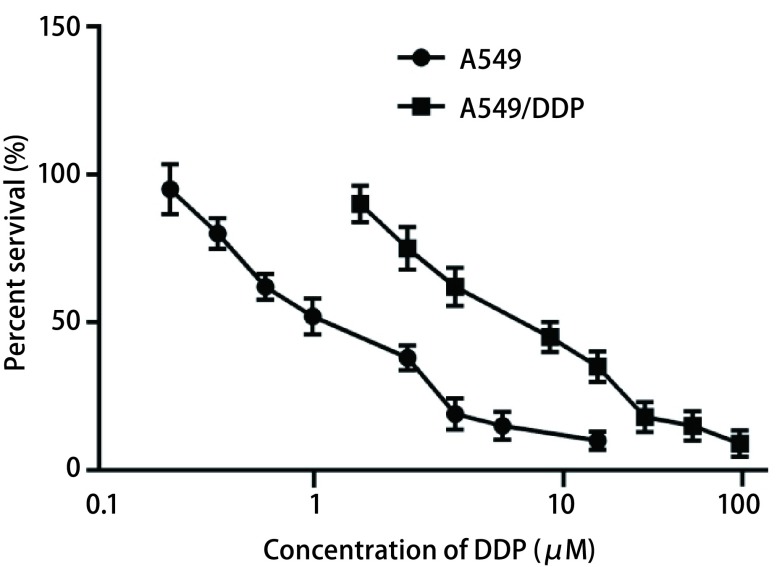
A549细胞与A549/DDP细胞对顺铂的耐药性 The resistance of A549 cells and A549/DDP cells to cisplatin

### 2.2 A549细胞与耐药A549/DDP细胞生长曲线比较

为验证构建顺铂耐药细胞株对A549细胞增殖的影响，本研究首先检测了A549细胞与耐药细胞株A549/DDP的生长曲线，如图 2显示，耐药A549/DDP细胞增值速度与其亲本细胞A549增殖速度几乎相同，曲线无显著差异（*P* > 0.05），说明构建耐药顺铂细胞株后未影响细胞的增殖能力。

**2:**
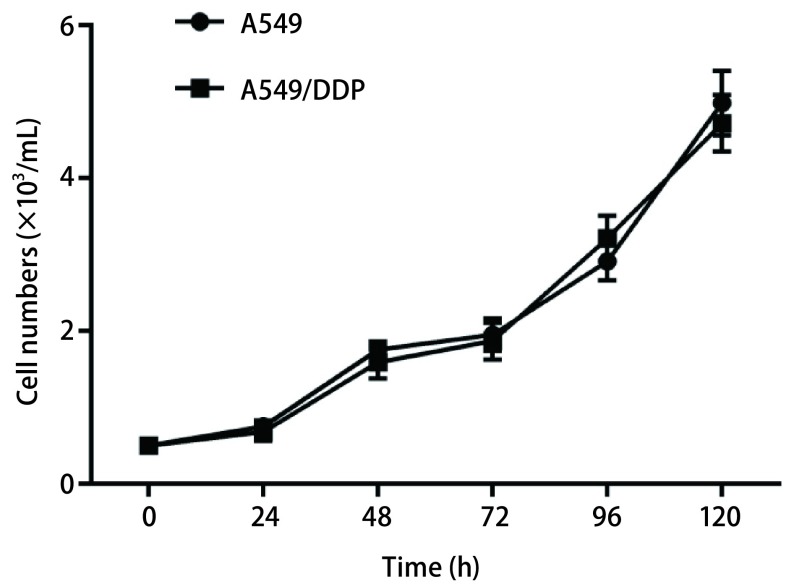
A549细胞与A549/DDP的细胞生长曲线 Cell growth curve of A549 cells and A549/DDP

### 2.3 A549和A549/DDP细胞中SOX4的表达

为验证SOX4与NSCLC细胞A549顺铂耐药的关系，本研究检测了耐药细胞株中SOX4蛋白的表达，结果如图 3显示，构建的耐药细胞株A549/DDP中SOX4的表达明显上调（*P* < 0.001），提示SOX4可能在NSCLC细胞耐药过程中发挥作用。

**3:**
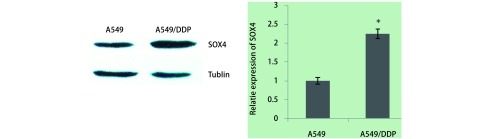
SOX4在A549与A549/DDP细胞中的表达 Expression of SOX4 in A549 and A549/DDP cells

### 2.4 敲减A549/DDP细胞中SOX4的表达对细胞耐药性的影响

为验证SOX4对NSCLC细胞A549顺铂耐药的影响，本研究在敲减SOX4后用CCK8法检测了A549/DDP细胞对顺铂的耐药性。结果如图 4显示，与转染对照siRNA con细胞比，siRNA转染在转录水平和蛋白表达水平均显著抑制了SOX4的表达（*P* < 0.001），而A549中SOX4的表达也较A549/DDP显著降低（*P* < 0.01）。通过细胞对于顺铂耐药性结果显示，敲减SOX4后，细胞对顺铂耐药性显著降低（*P* < 0.001），且在SOX4低表达的A549中，对于顺铂的耐药性同敲减了SOX4后耐药性相似。A549、A549/DDP、siRNA con、siRNA-1、siRNA-2的IC_50_分别为（1.98±0.24）、（28.17±4.36）、（26.57±6.21）、（0.87±0.28）、（0.92±0.57）。

**4:**
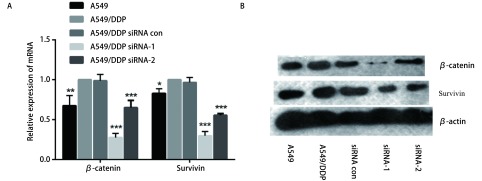
敲减SOX4后A549/DDP细胞对顺铂的耐药性。A：敲减SOX4后，实时定量PCR检测SOX4 mRNA表达水平；B：敲减SOX4后，Western bot检测蛋白表达水平；C：检测A549及不同敲减SOX4的A549/DDP细胞对顺铂的耐药性。 Resistance of A549/DDP cells to cisplatin after knockdown of SOX4. A: Expression level of SOX4 mRNA of A549/DDP after knockdown of SOX4 was detected by real-time quantitative PCR; B: The expression level of SOX4 of A549/DDP after knockdown of SOX4 was detected by Western blot; C: Resistance of A549 and A549/DDP cells to cisplatin.

### 2.5 敲减SOX4对Wnt信号通路相关蛋白表达的影响

为探索SOX4对NSCLC细胞对顺铂耐药性的分子机制，本研究检测了敲减SOX4后Wnt信号通路关键蛋白β-catenin及其下游靶基因Survivin的mRNA和蛋白表达情况，结果如图 5所示，敲减SOX4后，A549/DDP细胞内β-catenin及Survivin的mRNA水平显著降低（*P* < 0.01），同时Western blot结果显示两种蛋白的表达显著降低。该结果显示SOX4影响了β-catenin与Survivin蛋白的表达，可能与细胞对于顺铂耐药性改变的因素。

**5:**
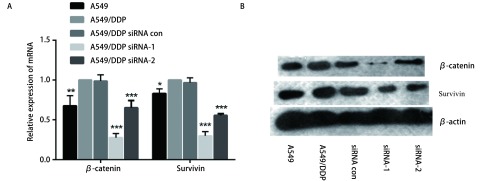
敲减SOX4后对*β*-catenin和Survivin表达的影响。A：敲减SOX4后，实时定量PCR检测*β*-catenin和Survivin mRNA表达水平；B：敲减SOX4后，Western blot检测*β*-catenin和Survivin蛋白表达。 Expression of *β*-catenin and Survivin of A549/DDP after knockdown of SOX4. A: Expression level of *β*-catenin mRNA and Survivin mRNA of A549/DDP after knockdown of SOX4 was detected by real-time quantitative PCR; B: Expression of *β*-catenin and Survivin of A549/DDP after knockdown of SOX4 was detected by Western blot.

## 3 讨论

肺癌是目前全球范围内恶性肿瘤之一，NSCLC是最常见的肺癌类型，也是肺癌治疗的重点^[9]^，化疗是重要的临床治疗手段，以顺铂（DDP）为主的联合化疗也是目前治疗晚期NSCLC的标准方案，对顺铂耐受是导致化疗失败的主要原因。因此，本研究首先构建了顺铂耐药细胞株A549/DDP作为研究模型，探究NSCLC对顺铂的耐药机制。

研究^[10, 11]^表明，SOX4在多种癌症中均有异常表达，其表达的增加可作为乳腺癌和NSCLC患者不良预后的生物标志物，并在一定程度上影响了相关肿瘤细胞对药物的敏感性。但SOX4与NSCLC对顺铂耐药性的影响目前无相关报道。本研究发现，顺铂耐药细胞株A549/DDP中SOX4蛋白的表达水平显著高于其亲本A549细胞，并且siRNA敲减SOX4的表达后，A549/DDP细胞的耐药性显著降低，说明SOX4对NSCLC细胞对顺铂的耐药性有重要的调控作用，提示SOX4可作为增加NSCLC顺铂敏感性的重要靶点。

WNT/β-catenin信号通路在肿瘤发生发展过程中有重要的调控作用，Wnt信号通路的异常与多种肿瘤化疗耐受密切相关^[12, 13]^。研究表明，SOX4能够通过Wnt/β-catenin信号通路影响黑色素瘤细胞的增殖^[14]^，并且，Wnt信号通路下游靶分子Survivin与肿瘤抗药相关，其siRNA可能作为克服肿瘤抗药的新型治疗手段^[15]^。本研究发现，在耐药细胞株中干扰SOX4的表达后，Wnt信号通路关键蛋白β-catenin及其下游靶分子Survivin的蛋白表达水平显著降低。由此推测，SOX4可能通过Wnt信号通路中β-catenin的表达进而调节其下游靶分子Survivin从而介导A549/DDP细胞对顺铂耐药。

综上所述，本研究利用顺铂诱导构建耐药细胞株A549/DDP，发现其SOX4的蛋白表达水平显著高于亲本细胞，进一步研究表明，敲减SOX4后，A549/DDP细胞耐药性显著降低，且Wnt信号通路关键蛋白β-catenin及其下游靶分子Survivin的蛋白表达水平显著降低，推测SOX4可能通过调节Wnt信号通路影响NSCLC的敏感性，具体分子机制还有待进一步研究，能否将SOX4作为克服NSCLC耐药的新靶点也有待进一步的探索。
